# Postmastectomy Radiotherapy for Locally Advanced Breast Cancer Receiving Neoadjuvant Chemotherapy

**DOI:** 10.1155/2014/719175

**Published:** 2014-06-22

**Authors:** Icro Meattini, Sara Cecchini, Vanessa Di Cataldo, Calogero Saieva, Giulio Francolini, Vieri Scotti, Pierluigi Bonomo, Monica Mangoni, Daniela Greto, Jacopo Nori, Lorenzo Orzalesi, Donato Casella, Roberta Simoncini, Massimiliano Fambrini, Simonetta Bianchi, Lorenzo Livi

**Affiliations:** ^1^Department of Radiation-Oncology, University of Florence, Largo G. A. Brambilla 3, 50134 Florence, Italy; ^2^Molecular and Nutritional Epidemiology Unit, ISPO (Cancer Research and Prevention Institute), University of Florence, Largo G. A. Brambilla 3, 50134 Florence, Italy; ^3^Diagnostic Senology Unit, University of Florence, Largo G. A. Brambilla 3, 50134 Florence, Italy; ^4^Department of Surgery, University of Florence, Largo G. A. Brambilla 3, 50134 Florence, Italy; ^5^Department of Gynecology and Obstetrics, University of Florence, Largo G. A. Brambilla 3, 50134 Florence, Italy; ^6^Department of Pathology, University of Florence, Largo G. A. Brambilla 3, 50134 Florence, Italy

## Abstract

Neoadjuvant chemotherapy (NAC) is widely used in locally advanced breast cancer (BC) treatment. The role of postmastectomy radiotherapy (PMRT) after NAC is strongly debated. The aim of our analysis was to identify major prognostic factors in a single-center series, with emphasis on PMRT. From 1997 to 2011, 170 patients were treated with NAC and mastectomy at our center; 98 cases (57.6%) underwent PMRT and 72 cases (42.4%) did not receive radiation. At a median follow-up period of 7.7 years (range 2–16) for the whole cohort, median time to locoregional recurrence (LRR) was 3.3 years (range 0.7–12.4). The 5-year and 10-year actuarial LRR rate were 14.5% and 15.9%, respectively. At the multivariate analysis the factors that significantly correlated with survival outcome were ≥4 positive nodes (HR 5.0, 1.51–16.52; *P* = 0.035), extracapsular extension (HR 2.18, 1.37–3.46; *P* = 0.009), and estrogen receptor positive disease (HR 0.57, 0.36–0.90; *P* = 0.003). Concerning LRR according to use of radiation, PMRT reduced LRR for patient with clinical T3 staged disease (P = 0.015). Our experience confirmed the impact of pathological nodal involvement on survival outcome. PMRT was found to improve local control in patients presenting with clinical T3 tumors, regardless of the response to chemotherapy.

## 1. Introduction

Neoadjuvant chemotherapy (NAC) is widely used in locally advanced breast cancer (BC) treatment. It is increasingly used in women with early stage disease [1]. It allows the clinicians to observe tumor response and modify radiotherapy plan [2]. Adjuvant therapeutic strategies for patients who underwent NAC do not differ substantially from patients treated with upfront surgery [3–6]; nevertheless, the role of postmastectomy radiotherapy (PMRT) after NAC is strongly debated. Moreover there is a lack of prospective trials in this treatment setting.

In an era of “tailored treatment,” additional data are needed for patients who receive this treatment sequence to determine which subsets of patients can benefit from radiation [7].

The aim of our analysis was to identify major prognostic factors in a single-center series of advanced BC patients receiving NAC, with emphasis on the role of PMRT.

## 2. Materials and Methods

### 2.1. Patient Population

From 1997 to 2011, 226 patients were treated with NAC and mastectomy at the University of Florence Radiotherapy Unit (Florence, Italy). Previous solid tumors, age less than 18, and BC recurrences or contralateral tumor were considered exclusion criteria of the study. To minimize bias, all patients with disease recurrence within 2 months after surgery, completion of adjuvant chemotherapy, and a minimum follow-up period shorter than 6 months were excluded from analysis. We retrospectively reviewed a series of 170 BC patients who received NAC and mastectomy; 98 cases (57.6%) underwent PMRT and 72 cases (42.4%) did not receive radiation. Informed consent was obtained from all patients.

In our multidisciplinary team, specialized expert pathologists, dedicated to BC specimens' evaluation, perform pathology assessment. Estrogen receptor (ER) status, progesterone receptor (PgR) status, and Ki-67 labeling index determined with the MIB1 monoclonal antibody were assessed. For ER and PgR status two categories (negative/positive) were considered according to well-established cut-off values [8]. HER2 immunohistochemistry (IHC) scores of 0 and 1+ were considered negative. HER2 IHC 3+ and fluorescent in situ hybridization (FISH)—amplified tumors, were considered positive. All IHC 2+ tumors were tested for gene amplification by FISH. The applied well-validated [9] primary antibodies for evaluating ER and PgR in BC by IHC have been extensively described in earlier published reports [10, 11].

BC was classified according to the histological type and the AJCC TNM classification of malignant tumors. Concerning Ki-67, we used a validated [12] cut-off value of 20% to distinguish Ki-67 high from Ki-67 low, although the ideal threshold has not been identified yet and varies widely from 1 to 28.6% [13].

### 2.2. Treatment Details

All patients, except 8 cases, received anthracyclines as part of a combination chemotherapy regimen (98.8%), with 69 patients (40.6%) also receiving a taxane. Concerning HER2 status, 23 patients had HER2 positive and 45 patients had HER2 negative status at pathological specimen; in 102 cases HER2 status was undetermined or missing. None of these patients were treated with neoadjuvant or adjuvant trastuzumab, since they were treated before 2006.

Most commonly administered chemotherapy regimens were FEC and ET; FEC chemotherapy consisted of 500 mg/m^2^ 5-fluorouracil, 75 mg/m^2^ epirubicin, and 500 mg/m^2^ cyclophosphamide, given on day 1. The ET regimen consisted of 75 mg/m^2^ epirubicin and 75 mg/m^2^ docetaxel, given on day 1. The median number of chemotherapy cycles received was 4 (mean, 4.7; range, 2–6).

Additionally, 108 patients (88.2%) received adjuvant hormonal therapy: tamoxifen for 5 years (*n* = 63; 37.1%), aromatase inhibitors for 5 years (*n* = 52; 30.6%), and tamoxifen for 2 years and then shift to aromatase inhibitors (*n* = 35; 20.5%).

Concerning PMRT, the treatment volumes typically included the chest wall and draining lymphatics (*n* = 84; 85.7%), consisting in the supraclavicular (SCV) and infraclavicular (ICV) nodal region (total dose 50 Gy; 2 Gy daily fractions), with mixed photon and electron beams technique, chosen at physician discretion. In our Institute we did not irradiate mammary internal nodal region, unless pathologically involved. In selected cases (*n* = 14; 14.3%) only chest wall was irradiated.

Patients underwent a treatment-planning noncontrast CT scan. Concerning CTV identification, for chest wall volume, the cranial limit was the caudal border of the clavicular head, the caudal limit was the contralateral inframammary fold, the lateral limit was the midaxillary line, and the medial limit was the sterna-rib junction. For SCV nodes the cranial limit was a line passing below the cricoid cartilage, the caudal limit was the caudal edge of the clavicular head, the anterior limit was the poster edge of the sternocleidomastoid (SCM) muscle, the posterior limit was the anterior aspect of the scalene muscle, the lateral limit was the lateral edge of the SCM muscle cranially, and the junction first rib-clavicle caudally, and the medial limit was a line excluding thyroid and trachea. For ICV nodes, the cranial limit was pectoralis minor muscle insert on coracoid, the caudal limit was axillary vessels cross-medial edge of pectoralis minor muscle, the anterior limit was the posterior surface of pectoralis major muscle, the posterior limit was ribs and intercostal muscles, the lateral limit was the medial border of pectoralis minor muscle, and the medial limit was the thoracic inlet.

### 2.3. Statistical Analysis

For the survival analysis, the date of histological BC diagnosis was used as the start of observation. The survival time was calculated from the date of diagnosis to the date of death or the date of the last follow-up for living patients. We considered as events the deaths for all causes (overall survival, OS). We also estimated the disease-free survival (DFS) as the interval time from the date of diagnosis to the date of locoregional recurrence (LRR) or distant metastases (DM).

The actuarial rates of death, LRR, or DM were calculated according to the Kaplan-Meier method, and comparisons were made using the log-rank test. Estimated relative risk of death, LRR, or DM were expressed as hazard ratios (HR) and their corresponding 95% confidence intervals (95% CI).

The clinical and pathologic factors that were statistically significant (two-tailed *P* < 0.05) on univariate analysis of LRR, DM, or OS were included in a multivariate analysis using the Cox proportional hazards regression model. All statistical tests were performed by the SAS software (SAS for Windows, version 9.1).

In order to analyze if the concomitant presence of well-known [14] risk factors influences the LRR rate, we stratified the patients in three risk groups (0-1 factors versus 2 factors versus 3–5 factors). We considered the following risk features: skin/nipple involvement, SCV nodal involvement, no tamoxifen use, extracapsular extension (ECE), and ER negative disease.

## 3. Results

### 3.1. Series Characteristics

The median age at BC diagnosis was 48.9 years (range 24–76). The median follow-up periods of all irradiated and nonirradiated cases were 7.2 and 6.7 years, respectively.

Table 1 showed major clinical characteristics of the whole series and stratified by radiation treatment. When compared with patients who did not receive PMRT, a larger number of irradiated patients had greater clinical and pathological T, N, and combined AJCC TNM stage (*P* ≤ 0.024 for all comparisons). There were no differences between the two groups considering age, histology, nuclear grade, lymph vascular invasion (LVI), downstaging after NAC based on pathological response, use of hormonal therapy, Ki-67 index, and percentage of ER and PgR.

### 3.2. Prognostic Factors of the Whole Series

At a median follow-up period of 7.7 years (range 2–16; standard deviation (SD) 5.1), 98 patients are alive (57.6%) and 72 patients are dead (42.4%). Median time to LRR (*n* = 26) was 3.3 years (range 0.7–14.6; SD 3.9); median time to DM (*n* = 86) was 3.0 years (range 0.7–12.4; SD 2.6). The 5-year and 10-year actuarial LRR rate were 14.5% and 15.9%, respectively.

The majority of LRR failures occurred on the chest wall (*n* = 15; 57.7%). SCV was the first nodal site of relapse in 7 cases (26.9%). Axillary (*n* = 2), infraclavicular (*n* = 1), and internal mammary nodal regions (*n* = 1) were rare sites of LRR (15.4%).

Table 2 showed LRR, DM, and OS rates according to main clinical features. In Table 3 survival rates were summarized according to major pathologic characteristics.

The factors that significantly correlated with poor LRR outcome were clinical N2 tumors, pathologic skin involvement, LVI, and the presence of ECE. The factors that significantly correlated with poor DM outcome were clinical N2 tumors, pN2, pN3 tumors, LVI, and ECE. Concerning OS, the significant protective features were pT1 tumors and ER positive status, while pN1, pN2, pN3 tumors, and ECE were unfavorable risk factors.

In the multivariate Cox regression analysis no factors were independently associated with LRR. The multivariate analysis of distant metastases occurrence and overall survival are described in Table 4. The factors that significantly correlated with survival outcome were ≥4 positive nodes, ECE, and estrogen receptor positive disease.

**Figure 1:**
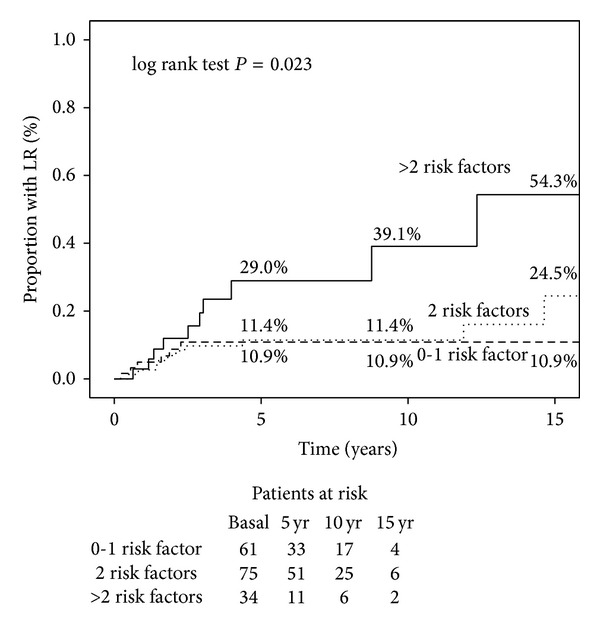
Locoregional recurrence rates according to number of selected risk factors.

The LRR rate for the 61 patients (35.9%) with one or none of selected [14] risk factors (6 events) was 10.9%, the 75 patients (44.1%) with two factors (10 events) had a rate of 24.5%, and the 34 patients (20%) with three or more factors (10 events) had a rate of 54.3% (log rank test *P* = 0.023; Figure 1). In an analysis stratified by radiation use, PMRT showed a protective effect (*P* = 0.029).

### 3.3. Locoregional Recurrence Rate according to Use of Postmastectomy Radiotherapy

In Table 5 the impact of PMRT on LRR for various subgroups of patients is shown.

**Figure 2:**
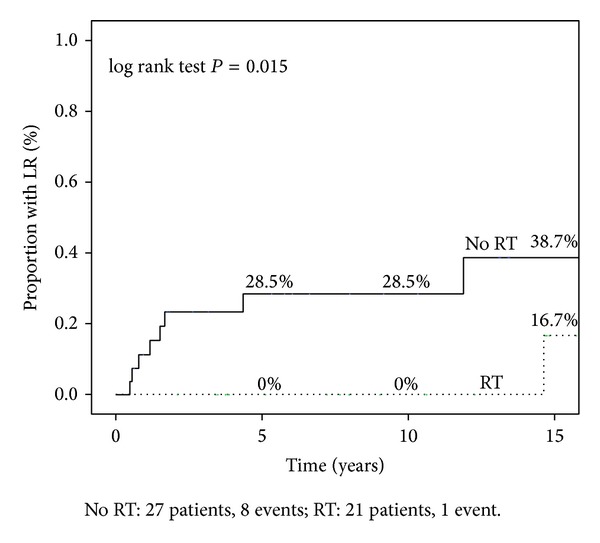
Kaplan-Meier survival curve of locoregional recurrence for the cohort of patients with clinically T3 disease who received NAC and mastectomy. Patients were stratified by whether they received postmastectomy radiation (PMRT; *n* = 21) or not (No PMRT; *n* = 27). Statistical comparison between the survival curves was made using the log-rank test (*P* = 0.015).

PMRT was associated with reduced LRR for patient with clinical T3 staged disease (16.7% versus 38.7%; *P* = 0.015; Figure 2). For patients with clinical T4 and clinical N2 and N3 tumors, no difference in LRR rates was observed. Also concerning pathological features, no difference in LRR rates was observed. In addition, in the subset of patients that achieved complete response after NAC (pCR; *P* = 0.29) or downstaging (*P* = 0.68), no statistical significance was evidenced.

### 3.4. Treatment Safety

Major hematological and nonhematological side effects are summarized in Table 6. The most represented hematological G3–G5 side effect was neutropenia (17%). The most frequent radiotherapy-related acute side effect was erythema (33%). At a median follow-up of 7.2 years, the most represented late RT-related side effect was fibrosis (20%).

## 4. Discussion

The Danish and the British Columbia trials have established the survival advantage following radiotherapy in postmastectomy patients [15, 16]. The Early Breast Cancer Trialists' Collaborative Group meta-analysis has demonstrated that PMRT, besides improving local control rates, confers an OS benefit [17]. On the basis of these studies and others, the role of the indication to PMRT has traditionally been determined by pathologic staging, with surgery as the first treatment modality [18, 19].

NAC is nowadays based on regimens containing anthracyclines, taxanes, and trastuzumab in case of HER2 positive disease. The toxicity profile of these drugs is well known, namely, characterized by potential cardiac [20–23] and pulmonary [24–26] adverse events. Although NAC has many advantages, its impact on surgical staging reduces the applicability of the traditional pathologic guidelines for PMRT.

Guidelines for the use of PMRT after NAC have not been established. Retrospective series have demonstrated that the omission of PMRT after NAC in high-risk patients can result in an unacceptable high rate of LRR, even in case of pathological complete response [7, 27]. For this reason, the role of PMRT is generally determined by clinical staging before NAC without regard for the response to NAC [18].

Risk factors for LRR in this specific setting are not well established. Advanced clinical or pathologic stage, triple-negative receptor status, and presence of LVI and/or ECE emerged as high-risk features that should warrant consideration of PMRT after NAC [28]. In our experience greater clinical nodal status, tumor stage, the presence of LVI, and nodal ECE emerged as adverse prognostic factors; these results are in line with many published series [7, 18, 28, 29].

Concerning age at diagnosis, Garg et al. [30] retrospectively analyzed 107 consecutive BC patients younger than 35 years with stage IIA–IIIC disease, treated with doxorubicin-based NAC and mastectomy, with or without PMRT. In this experience the use of PMRT led to a statistically greater rate of local control and OS compared with patients without PMRT.

Response to NAC is another debated issue in adjuvant PMRT decision-making. Data regarding LRR rates in patients who achieve a pCR are limited, although few data supported stage IIIA patients with pCR as being at low risk [28]. Concerning tumor biology and chemotherapy response, many experiences showed that residual disease after NAC seems to have a greater implication for outcome for those in whom systemic therapy would have been expected to produce a more favorable response, such as ER and HER2 positive patients [31–34].

Conversely, other studies suggested how PMRT should be indicated regardless of response to NAC [18, 35]. Also in our series disease downstaging and/or pCR to NAC were not independent prognostic factors for LRR occurrence.

In our experience PMRT was significantly protective only in case of clinical staged T3 tumors, regardless of response to NAC. Our results are consistent with the experience of M. D. Anderson Cancer Center, which in our knowledge represents the largest published series.

In a relevant study published in 2004, Huang et al. [7] showed how radiation was found to benefit both local control and survival for patients presenting with clinical T3 tumors or stage III-IV disease (ipsilateral SCV nodal) and for patients with four or more positive nodes.

In 2011, Nagar et al. [36] tried to determine the impact of PMRT after NAC on LRR in 162 patients with clinical T3N0 disease. PMRT was effective in reducing the LRR rate, even when there was no pathologic evidence of nodal involvement after NAC.

However we are aware of the limitations of our retrospective study: the two cohorts in the analyses had differences in several factors, and the more advanced tumor characteristics were in the PMRT group. PMRT may overcome negative pathologic features in the cohort.

A complex evaluation based on the presence of multiple risk factors should be of primary importance in the decision-making process for PMRT after NAC.

Fowble et al. [28] identified a cohort of women treated with NAC and mastectomy for whom PMRT may be omitted according to the projected risk of LRR. Seven breast cancer physicians from the University of California cancer centers created 14 hypothetical clinical case scenarios; an overall summary risk assessment table was developed, using the American College of Radiology rating scale. Clinical stage II (T1-2N0-1) patients, aged > 40 years, with ER positive subtype, with pCR or 0–3 positive nodes without LVI or ECE, were identified as having <10% risk of LRR without radiation.

Huang et al. [7, 14] retrospectively reviewed the hospital records of 542 patients treated on six consecutive institutional prospective trials using NAC and PMRT. In the multivariate analysis, skin/nipple involvement, SCV nodal involvement, no tamoxifen use, ECE, and ER negative were independently associated with developing LRR (HR 2.1–2.8; *P* < 0.001–0.020). The 10-year rate of LRR for patients with one or none of these factors was only 4%, but patients with two factors had a rate of 8%, and patients with three or more factors had a rate of 28% (*P* < 0.0001 for 0-1 factor versus 3–5 factors).

In order to validate the independent factors shown in the experience of the M. D. Anderson Cancer Center, we performed the same multiple factors analysis. Also in our series we found a significant higher LRR rate in patients with a greater number of risk factors (HR 2.70; 95% CI 1.12–6.53; *P* = 0.023; 54.3% LRR rate for patients with 3–5 factors).

The 2007 National Cancer Institute conference report recommended PMRT after NAC for patients presenting with clinical stage III disease or those with positive nodes after chemotherapy [37]. Our experience adds strength to the experiences that suggest PMRT after NAC based on clinical staging; however, we strongly believe that PMRT after NAC should be indicated following a comprehensive assessment of multiple factors.

## 5. Conclusions

Our experience confirmed the impact of pathological nodal involvement in patients' outcome. After NAC and mastectomy, PMRT was found to benefit local control of patients presenting with clinical T3 tumors, regardless of the response to chemotherapy. Radiation should always be considered after a careful multidisciplinary assessment of multiple risk factors. However prospective trials in properly selected patients are strongly needed.

## Figures and Tables

**Table 1:** Distribution of 170 breast cancer cases according to adjuvant radiotherapy.

Feature	Total *n* = 170	No PMRT *n* = 72	PMRT *n* = 98	*P* value°
Age groups				
≤40	33	18	15	
41–50	62	25	37	
51–60	5	18	32	
>60	25	11	14	0.40
Clinical T classification				
T1	4	2	2	
T2	52	28	24	
T3	48	27	21	
T4	66	15	51	**0.002**
Clinical N classification				
N0	42	20	22	
N1	95	45	50	
N2	33	7	25	**0.024**
Clinical stage				
IIA	16	8	8	
IIB	51	32	19	
IIIA	35	17	18	
IIIB	63	15	48	
IIIC	3	—	5	**0.0001**
Multiple Foci				
No	99	50	49	
Yes	71	2	49	**0.012**
Pathologic T classification				
pTx/pTis	14	7	7	
pT1	26	13	13	
pT2	75	39	36	
pT3	18	7	11	
pT4	37	6	31	**0.007**
Pathologic N classification				
pN0	20	14	6	
pN1	63	29	34	
pN2	52	16	36	
pN3	35	13	22	**0.02**
Downstage				
No	115	43	72	
Yes	55	29	26	0.07
Histology				
Ductal invasive	118	55	63	
Lobular invasive	36	12	24	
Others	16	5	11	0.24
Pathologic skin involvement				
Absent	136	66	70	
Present	34	6	28	**0.001**
Extracapsular extension				
Absent	115	52	63	
Present	55	20	35	0.32
LVI				
Absent	101	47	54	
Present	69	25	44	0.21
Nuclear grading∗				
G1	11	6	5	
G2	54	26	28	
G3	80	33	47	0.58
Ki 67 index				
<20	117	53	64	
≥20	53	19	34	0.32
ER status				
Negative	64	30	34	
Positive	106	42	64	0.42
PgR status				
Negative	83	40	43	
Positive	87	32	55	0.16
NAC regimen				
Anthracyclines-based	69	27	42	
Anthracyclines and taxanes-based	93	44	49	
No anthracyclines	8	1	7	0.13
Adjuvant hormonal therapy				
No	62	28	34	
Tamoxifen	75	31	44	
AIs	33	13	20	0.89

*Some data are missing; °*P* value from Fisher exact test or chi-square for trend, as appropriate.

PMRT: postmastectomy radiotherapy; LVI: lymphovascular invasion; ER: estrogen receptors; PgR: progesterone receptors; NAC: neoadjuvant chemotherapy; AIs: aromatase inhibitors.

**Table 2:** Locoregional recurrence, distant metastases, and overall survival rates according to clinical factors.

Variable	Patient	Overall survival		LRR free survival		DM free survival	
		Deaths	*P* value	LRR	*P* value	DM	*P* value
Age groups							
≤40	33	16		6		20	
41–50	62	24		9		20	
51–60	5	20		9		25	
>60	25	12	0.73	2	0.74	13	0.67
Clinical T							
T1	4	3		0		3	
T2	52	15		3		18	
T3	48	17		9		25	
T4	66	37	0.09	14	0.28	40	0.12
Clinical N							
N0	42	14		5		15	
N1	95	39		11		49	
N2	33	19	0.15	10	**0.034**	22	**0.028**
Clinical stage							
IIA	16	5		0		5	
IIB	51	17		6		21	
IIIA	35	13		6		20	
IIIB	63	36		14		39	
IIIC	3	1	0.15	0	0.19	1	0.09
Multiple Foci							
No	99	40		18		46	
Yes	71	32	0.60	8	0.28	40	0.19
Downstage							
No	115	50		19		64	
Yes	55	22	0.34	7	0.31	22	**0.029**
PMRT							
No	72	31		12		36	
Yes	98	41	0.83	14	0.57	50	0.93
PMRT volumes							
Chest wall	14	5		1		5	
Chest wall + SC	74	34	0.35	12	0.38	43	0.11
NAC regimen							
Anthracyclines-based	69	35		14		41	
Anthracyclines- and taxanes-based	93	33		10		41	
No anthracyclines	8	4	0.25	2	0.29	4	0.16
Adjuvant hormonal therapy							
No	62	34		10		35	
Tamoxifen	75	28		11		34	
AIs	33	10	0.11	5	0.93	17	0.31

Total	170	72		26		86	

LRR: locoregional recurrence; DM: distant metastases; PMRT: postmastectomy radiotherapy; NAC: neoadjuvant chemotherapy; SC: supraclavicular nodal region; AIs: aromatase inhibitors.

**Table 3:** Locoregional recurrence, distant metastases, and overall survival rates according to pathologic factors.

Variable	Patient	Overall survival		LRR free survival		DM free survival	
		Deaths	*P* value	LRR	*P* value	DM	*P* value
Multiple foci							
No	99	40		18		46	
Yes	71	32	0.60	8	0.28	40	0.19
Pathologic T							
pTx/pTis	14	8		3		8	
pT1	26	5		0		7	
pT2	75	29		8		38	
pT3	18	10		5		11	
pT4	37	20	**0.04**	10	**0.015**	22	0.13
Pathologic N							
pN0	20	3		2		3	
pN1	63	27		9		28	
pN2	52	26		12		33	
pN3	35	16	**0.026**	3	0.12	22	**0.0002**
Stage							
0	3	2		1		2	
I	6	0		0		0	
IIa	25	7		3		7	
IIb	25	9		4		9	
IIIa	43	20		6		27	
IIIb	39	18		8		21	
IIIc	29	16	**0.049**	4	0.76	20	**0.0006**
Histology							
Ductal invasive	118	52		19		59	
Lobular invasive	36	13		1		19	
Others	16	7	0.85	6	**0.008**	8	0.94
Pathologic skin involvement							
Absent	136	56		16		67	
Present	34	16	0.78	10	**0.018**	19	0.83
LVI							
Absent	101	38		14		44	
Present	69	34	0.19	12	0.35	42	**0.044**
Nuclear grading∗							
G1	11	4		0		5	
G2	54	17		3		25	
G3	80	40	0.18	16	**0.024**	44	0.52
Ki 67 index							
<20	117	48		21		57	
≥20	53	24	0.15	5	0.34	29	0.09
ER status							
Negative	64	35		10		37	
Positive	106	37	**0.015**	16	0.99	49	0.20
PgR status							
Negative	83	36		13		41	
Positive	87	36	0.83	13	0.87	45	0.83
Extracapsular extension							
Absence	115	37		13		48	
Presence	55	35	**0.0007**	13	**0.035**	38	0.003

Total	170	72		26		86	

*Some data are missing.

LRR: locoregional recurrence; DM: distant metastases; PMRT: postmastectomy radiotherapy; LVI: lymph vascular invasion; ER: estrogen receptors; PgR: progesterone receptors.

**Table 4:** Multivariate analysis of distant metastases occurrence and overall survival.

Factor	Hazard ratio	95% confidence interval	*P* value
Distant metastases			
pN2	6.95	2.11–22.56	0.007
pN3	7.21	2.15–24.18	0.009
Extracapsular extension	1.91	1.25–2.92	0.023
Overall survival			
pN2	5.00	1.51–16.52	0.035
pN3	4.50	1.31–15.48	0.037
Extracapsular extension	2.18	1.37–3.46	0.009
Estrogen receptor positive disease	0.57	0.36–0.90	0.003

**Table 5:** Locoregional recurrence-free survival rate according to postmastectomy radiotherapy.

Feature	*N*	No PMRT	PMRT	LRR		*P* value
				No PMRT	PMRT	
Age groups						
≤40	33	18	15	5	1	0.13
41–50	62	25	37	4	5	0.53
51–60	5	18	32	3	6	0.62
>60	25	11	14	0	2	0.24
Clinical T						
T1	4	2	2	0	0	—
T2	52	26	24	3	0	0.12
T3	48	27	21	8	1	**0.015**
T4	66	15	51	1	13	0.15
Clinical N						
N0	42	20	22	3	2	0.64
N1	95	45	90	7	4	0.15
N2	33	7	26	2	8	0.87
Multiple foci						
No	99	50	49	1	7	0.27
Yes	71	22	49	1	7	0.26
Pathologic T						
pTx/pTis	14	7	7	2	1	0.29
pT1	26	13	13	0	0	—
pT2	75	39	36	6	3	0.10
pT3	18	7	11	3	2	0.18
pT4	37	6	31	1	9	0.49
Pathologic N						
pN0	20	14	6	2	0	0.35
pN1	63	29	34	3	6	0.62
pN2	52	16	36	5	7	0.16
pN3	35	13	22	2	1	0.54
Downstage						
No	115	43	72	8	11	0.51
Yes	55	29	26	4	3	0.68
Histology						
Ductal invasive	118	55	63	9	10	0.78
Lobular invasive	36	12	24	0	1	0.48
Others	16	5	11	3	3	0.17
Pathologic skin involvement						
Absent	136	66	70	11	5	0.061
Present	34	6	28	1	9	0.44
LVI						
Absent	101	47	54	8	6	0.32
Present	69	25	44	4	8	0.93
Nuclear grading∗						
G1	11	6	5	0	0	—
G2	54	26	28	3	0	0.06
G3	80	33	47	7	9	0.86
Ki 67 index						
<20	117	53	64	10	11	0.76
≥20	53	19	34	2	3	0.69
ER status						
Negative	64	30	34	4	6	0.81
Positive	106	42	64	8	8	0.32
PgR status						
Negative	83	40	43	6	7	0.93
Positive	87	32	65	6	7	0.39
Extracapsular extension						
Absence	115	52	63	8	5	0.20
Presence	55	20	35	4	9	0.89
NAC regimen						
Anthracyclines-based	69	27	42	5	9	0.74
Anthracyclines- and taxanes-based	93	44	49	6	4	0.38
No anthracyclines-based	8	1	7	1	1	**0.008**
Adjuvant hormonal therapy						
No	62	28	34	4	6	0.86
Tamoxifen	75	31	44	5	6	0.71
AIs	33	13	20	3	2	0.32

Total	170	72	98	12	14	

*Some data are missing. LRR: locoregional recurrence; PMRT: postmastectomy radiotherapy; LVI: lymph vascular invasion; ER: estrogen receptors; PgR: progesterone receptors; NAC: neoadjuvant chemotherapy; AIs: aromatase inhibitors.

**Table 6:** Main chemotherapy- and radiotherapy-related adverse events.

	*N*	%
*Chemotherapy-related side effects *		
Anemia		
Grades 0–2	150	88
Grades 3–5	20	12
Neutropenia		
Grades 0–2	142	83
Grades 3–5	28	17
Piastrinopenia		
Grades 0–2	150	88
Grades 3–5	20	20
Mucositis		
Grades 0–2	158	93
Grades 3–5	12	7
Alopecia	162	95
Hypertransaminasemia	15	9
Febrile neutropenia	5	3
*Ra* *di* *ot* *he* *ra* *py*-*related* *side* *effects*°		
Erythema	32	33
Thoracic wall pain	25	25
Edema	3	3
Fatigue	30	30
Fibrosis	20	20
Telangiectasia	8	8

°PMRT group, any grades.
